# The Potential Environmental and Social Influence of the Inorganic Salt Hydrates Used as a Phase Change Material for Thermal Energy Storage in Solar Installations

**DOI:** 10.3390/ijerph20021331

**Published:** 2023-01-11

**Authors:** Edyta Nartowska, Marta Styś-Maniara, Tomasz Kozłowski

**Affiliations:** 1 Department of Geotechnics and Waste Management, Kielce University of Technology, 25-314 Kielce, Poland; 2 Department of Building Physics and Renewable Energy, Kielce University of Technology, 25-314 Kielce, Poland

**Keywords:** inorganic salt hydrates, environmental, public health, social, supercooling, phase separation, corrosiveness

## Abstract

The aim of this article is to assess the potential impact of inorganic salt hydrates used as PCM material in solar installations on the environment and human health and to assess the society’s approach to this technology. The properties of salt are discussed in two ways: first, by analyzing the environmental and health problems caused by chemical hazards on the basis of the available material safety data sheets. Secondly, by analyzing the potential disadvantages of salt hydrates in terms of environmental hazards based on the results of experimental studies available in the literature. Then, using questionnaires, the public approach to solar installations with a built-in converter containing salt hydrates is assessed. Disodium hydrogen phosphate dodecahydrate turned out to be the most prospective salt in terms of environmental, thermophysical, and economic properties for use in solar installations. Understanding the attitudes of the local community toward technologies using inorganic salt hydrates will enable appropriate action to be taken in the future to promote their development. Surveys have shown great public concern about their impact on the environment and human health. In this regard, it is necessary to implement information and promotion activities.

## 1. Introduction

Since the advent of renewable energy sources, they have been gaining greater and greater acceptance by society, especially when it comes to solar energy [1]. Local supply of this type of energy, with the use of distributed energy systems, plays an important role in the global infrastructure [2]. In some regions of the world, the potential environmental impact of different technologies is still little known [1]. On the one hand, replacing conventional sources (coal) with renewable energy has a positive effect on climate change and reduces soil acidification and eutrophication, but, on the other hand, technological advances and the introduction of new substances in installations, the impact of which is unknown in the long term, may cause serious environmental risk or social dissatisfaction [2]. The knowledge of the environmental effects of the interactions of various substances already at the stage of their selection for use in a photovoltaic installation in connection with their technical, economic, and social properties should be an important aspect in the process of designing such solutions [3,4].

Photovoltaics is the fastest growing field of energy generation. Photovoltaic panels absorb 80% of incident solar radiation, 20% of which is converted into electricity, and the rest is converted into heat, causing an increase in the temperature of the panel surface. The efficiency depends on several factors, such as: the material from which the panel is made, the intensity of the solar radiation, the surface temperature, the angle of the panel, and the wind speed. For each degree of temperature increase, the efficiency of the PV decreases by 0.4–0.5%. The panels operate most efficiently when they reach a nominal temperature close to 25 °C [4,5,6]. Scientists show that using the right phase change materials (PCM) will improve the performance of the photovoltaic (PV) panels. The storage of thermal energy in a photovoltaic system consists in the exchange in heat as a result of a phase change taking place in a suitable material and at an appropriate temperature. The thermal conductivity of the phase change materials is very low and ranges from 0.16 to 0.25 W/mK. Such an action lowers the surface temperature of the PV and increases the efficiency of the system [7].

One of the groups of phase change materials that are used in solar installations includes inorganic salts [8]. Inorganic salts, as opposed to organic ones, are considered more environmentally friendly and safe [9], have a higher volumetric latent heat, are cheaper, more easily available, their thermal conductivity is higher, they have lower volumetric changes, and they are a non-flammable material. However, the disadvantage is that they are corrosive, they are subject to overcooling, and the volume changes are high [8]. Taking care of the technical and economic aspects, there are works devoted to improving the properties of salt hydrates. One of the most promising trends is nanoencapsulation, which improves their structural properties and reduces costs and increases the potential of their applications [4,9,10]. Research is still being undertaken to create mixtures of salt hydrates that will lower their corrosiveness, as well as reduce or stabilize supercooling and volume changes [9,10,11,12]. Researchers are working on improving the environmental properties of PCMs. Elias and Stathopoulos, in their publication [4], wrote that energy efficiency is of key importance for maintaining the competitiveness and profitability of investments in solar systems. This, in turn, should take into account current or future environmental regulations. Despite the extensive literature on salt hydrates used as PCM materials in solar installations, little is known about the potential environmental effects of their use. Researchers usually only indicate that the environmental aspect is important [13,14,15,16], but there are no detailed studies. The present authors did not find any work that would analyze the technical, economic, environmental, and social aspects in the context of the use of non-organic salt hydrates for applications in solar installations. However, only full knowledge based on all these factors will allow a rational selection of appropriate salt hydrates and their mixtures and will enable sustainable development.

The article reviews, from the point of view of environmental risks, the eight considered to be the most promising inorganic salt hydrates used in solar installations [8,9,10,11,12]. They are magnesium chloride hexahydrate, magnesium nitrate hexahydrate, sodium sulfate decahydrate, sodium acetate trihydrate, sodium carbonate decahydrate, calcium chloride hexahydrate, disodium hydrogen phosphate dodecahydrate, and barium hydroxide octahydrate. The thermophysical properties of inorganic salt hydrates were presented, and the possibilities of their improvement were indicated. The costs of the substances as such have also been considered. Subsequently, the chemical risk of salt hydrates on the environment and humans was assessed. Then, the disadvantages of inorganic salt hydrates, such as supercooling, phase separation, and corrosiveness, were characterized, and the possibilities of its minimization were indicated. Corrosiveness may release substances into the environment. In turn, supercooling and phase separation can reduce the PV efficiency, which in turn increases the amount of waste. In the following part of the publication, the possibility of the utilization of the used salt hydrates was assessed. Then, with the help of questionnaires, the society’s approach to the solar installations enriched with salt converters was assessed. The basis of the questionnaires was to assess how important the environmental aspect is for people when choosing solar installations. As a result, the most technically, economically, and environmentally sustainable inorganic salt hydrate was selected for use in solar installations. Based on the literature review, the directions of future research, and the implementation of which will affect the sustainable development of the environment were indicated.

## 2. Thermophysical Parameters of the Salt and the Costs of Thermal Energy Storage

In phase change materials, such as salt hydrates, latent heat is stored, consisting of a phase change in the heat-storing material. During the change in the state of aggregation, a large amount of energy is accumulated, and the amount of heat exchanged is described by Formula (1).

*Q* = *m*[*c_ps_*(*T_m_* − *T_i_*) + *h_f_* + *c_pl_*(*T_f_* − *T_m_*)] (1)

where:

*m*—mass of the substance [kg],

*c_ps_*—specific heat of the solid [kJ/(kg∙K)],

*T_m_*—phase change temperature [K],

*T_i_*—initial temperature of the heat storage process [K],

*h_f_*—specific enthalpy of phase transition [kJ/kg],

*c_pl_*—specific heat of the liquid [kJ/(kg K)],

*T_f_*—final temperature of the heat storage process [K].

Most experiments with salt hydrates in photovoltaic systems focus on the latent heat absorption of the phase transition, i.e., the thermal energy absorption capacity for a solid-to-liquid phase transition of a substance. This is beneficial in temperature control, as salt hydrates assist in the passive cooling in photovoltaic modules [9].

Understanding the thermophysical parameters of the substances, supported by the results of experimental studies in solar installations, may prove helpful for the environmental assessment of these substances, which is described in the following sections. When analyzing the thermophysical parameters, it is advantageous if the substance accumulates large amounts of energy at a constant temperature or in a limited temperature range corresponding to the phase change in a given material. Moreover, the PCM phase change temperature should be within the temperature range of the given application. In turn, higher values of energy storage density translate into a smaller volume of material necessary to accumulate a given amount of heat, which may minimize the amount of waste [17]. The thermophysical parameters of the analyzed salts are presented below, based on the information contained in the safety data sheets and on the basis of experimental studies by various authors (Table 1).

Höhlein et al. [19] consider magnesium chloride hexahydrate a promising material for practical applications due to its good thermophysical properties. According to the authors of [19], the substance will also prove useful in waste heat transport systems. In turn, according to Saikrishnan et al. [36], the discussed hydrate is not the best for solar systems due to its low thermal conductivity. The authors analyzed the thermal performance of a solar-powered thermal energy storage (TES) system with MgCl_2_·6H_2_O. The material was sealed in copper cylindrical containers and placed vertically in the TES reservoir. The ability to store energy was assessed. Low thermal reactions of the components and low temperature changes in the morning were observed. The maximum efficiency of the solar collector was 72.5% between 1:00 p.m. and 2:00 p.m. and it decreased with the decrease in solar radiation.

Magnesium nitrate hexahydrate (MNH) has the appropriate phase transition temperature for use in solar thermal energy storage [37]. The thermophysical properties of MNH improve with an increase in the mass percentage of the carbon sphere as a filler. The optimal value is 0.5% by mass of the carbon material, and it improves the thermal conductivity of the MNH and the heat transfer coefficient. The authors obtained the best parameters for the composites with a carbon sphere and worse for nanographite [37]. The energy storage density is significantly improved when the eutectic substance, magnesium chloride hexahydrate, is used together with magnesium nitrate hexahydrate. However, low thermal conductivity limits the rate of heat uptake [38].

Li et al. [39] and Shein et al. [40] consider sodium sulfate decahydrate to be a suitable candidate for heat storage applications due to its high latent heat and desirable phase transition temperature, but it suffers from undercooling and phase separation. Similar limitations also exist with sodium acetate trihydrate (SAT) [9,24,41]. However, in the case of SAT, the high degree of supercooling and the high energy storage density make it an ideal flexible material for storing heat with almost no heat loss in both the short and long term, which provides enormous benefits for the energy system [42].

Rathold et al. [43] showed in their research that sodium carbonate decahydrate and calcium chloride hexahydrate are useful in thermal energy storage systems, but sodium carbonate decahydrate has a better efficiency of approximately 5–7% during charging and discharging. Pichandi et al. [44] created a eutectic mixture of sodium carbonate decahydrate with magnesium sulfate heptahydrate to improve the thermophysical properties of the PCM, including the latent heat of fusion and the phase transition temperature. In the PV-PCM system, an increase in the daily DC output power of 12.5% was observed as compared to the reference PV module.

Disodium hydrogen phosphate dodecahydrate (DHPD) is considered an excellent hydrated salt for storing latent heat due to its high latent heat and almost no phase separation. Its greatest disadvantages are supercooling and dehydration after exposure to air [28,45]. The leakage problem can be prevented by encapsulating it in porous expanded vermiculite (EV) [28]. DHPD is used in long-term solar heat storage systems where a higher storage efficiency is generated than in sensible heat storage systems. The efficiency of the system improves with the extension of the heat storage period [46].

Barium hydroxide octahydrate (BHO) is a salt with a high heat capacity but exhibiting a significant phenomenon of supercooling and phase separation during the cooling process, which limits its use. These problems can be minimized thanks to the use of nucleating and gelling agents [31,47]. Han et al. [48] increased the thermal conductivity of BHO by two to four times, creating composites with expanded graphite (EG), with little negative influence on other properties. The authors consider the material ideal for a solar energy storage system within finite thermal cycles.

Calcium chloride hexahydrate is compared by Pan et al. [49] to paraffinic PCM due to a high latent heat, thermal conductivity, and the possibility of storing excess heat. The substance melts at 29.6 °C, which can ensure the material melts when it absorbs excess heat from solar radiation. A serious problem is supercooling, which is minimized by means of nucleating agents, e.g., BaI_2_·6H_2_O, SrCl_2_·6H_2_O and SrBr_2_·6H_2_O [50]. In contrast, Donkres et al. [13] believe that calcium chloride hexahydrate does not meet the requirements for seasonal heat storage (domestic heating, domestic hot water). According to the authors [13], it is impossible to achieve the required temperatures during hydration with a reasonable energy storage density.

The literature review shows that the proposed salt hydrates are good candidates for applications as a PCM material in solar installations in terms of the thermophysical parameters. However, several authors point out the particular usefulness of SAT [42], BHO [48], and DHPD [28]. However, it should be borne in mind that each salt hydrate, apart from its advantages, has its limitations. Choosing the right substance is often a compromise. Methods are also sought to improve the thermophysical properties of the salt hydrates as outlined above. This is also confirmed by the authors of [10,51], who write that currently there is no material available that would meet all the requirements for commercial applications. Moreover, Song et. al. [10] indicate the limitations we must take into account when selecting a specific group of salt hydrates. According to the authors, nitrates have a low corrosivity, but also a low thermal conductivity, which can easily overheat. Carbonates have a low corrosivity and a high density and solubility, as well as a high melting point, but some break down easily and have a high viscosity. Chlorides, on the other hand, are corrosive.

Various authors have written about the relatively low cost of salt hydrates [25,30,42,46]. However, the prices of inorganic salt hydrates are compared to other PCM compounds and not to each other. It seems important to know the differences in the case of inorganic salt hydrates for the same type of application (solar installations), which may be important when choosing a given substance. The prices of the salt hydrates were determined on the basis of the price list available on the Sigma Aldrich website as of 01/07/2022 [35]. The most expensive salt hydrates are magnesium chloride hexahydrate, barium hydroxide octahydrate, magnesium nitrate hexahydrate, and sodium sulphate decahydrate. The cheapest ones are calcium chloride hexahydrate, disodium hydrogen phosphate dodecahydrate, and sodium carbonate decahydrate. The higher the density of a given salt hydrate, the smaller the volume of waste that can be generated in the future.

The most advantageous from an economic point of view, due to the relation of the price and density, are calcium chloride hexahydrate, in the first place, and disodium hydrogen phosphate dodecahydrate, in the second place. On the other hand, magnesium chloride hexahydrate and sodium acetate trihydrate seem to be the most unfavourable and can generate the largest volume of waste.

In order to ensure the conditions of the safe use of the equipment at high temperatures and under high pressure in terms of thermal energy storage, salt hydrates should meet a number of requirements. From the point of view of the environment, it is important to assess the chemical stability, toxicity, and flammability in the temperature range used [10]. Therefore, in the following chapters, the authors will discuss the environmental properties of the salt in two ways: first, by analyzing the environmental and health problems caused by chemical hazards on the basis of the available material safety data sheets. Secondly, by analyzing the potential disadvantages of the salt hydrates in terms of the environmental hazards based on the results of experimental studies available in the literature.

## 3. Assessment of Chemical Hazards of Inorganic Salt Hydrates for the Environment and Humans

This section discusses some of the health and environmental problems caused by the chemical hazards related to the potential salt hydrate toxicity, flammability, and explosiveness. The potentially harmful toxicity of a substance depends on many factors: chemical properties, the concentration of the substance, and the exposure time [14]. Most often, the main people exposed are the workers who assemble the system. However, in the event of a failure, exposure to the users and nearby residents cannot be ruled out. The easiest way of exposure of workers is the inhalation of vapours or dusts, as well as the direct contact in the event of a spill [14]. In the event of a failure and the unsealing of the installation, it is possible for pollutants to seep into the ground and groundwater, which may have a negative impact on various components of the environment. There is a clear warning on the safety data sheets to prevent salt hydrates from entering water, sewage, and soil.

To be environmentally friendly, salts should not exhibit a bioaccumulation capacity and should not be toxic to fish or daphnia in chronic action at a concentration of 1 mg/dm^3^, and, in prolonged exposure, >1 mg/dm^3^ [52]. They should not contain substances particularly harmful to the aquatic environment, causing water pollution [52]. Salt hydrates should not be classified as dangerous according to Regulation (EC) No 1272/2008 [53] on the classification, relating to labeling and packaging of substances and mixtures. In addition, the substances should be chemically stable under working conditions.

Based on the analysis of the safety data sheets of the eight salts in question, it was established that each of them is a chemically stable substance at room temperature, does not have the ability to bioaccumulate, is not toxic to the environment, is not dangerous within the meaning of Regulation (EC) No 1272/2008 [53], and is not carcinogenic to humans. The presented salt hydrates are non-combustible. In the case of magnesium nitrate, it should be noted that it is a strong oxidant that increases the flammability of other substances. The waste of six of the eight substances (except magnesium nitrate hexahydrate and disodium hydrogen phosphate dodecahydrate) should be treated as hazardous waste according to Directive 91/689/EC [54] of the European Union. Salt hydrates pose a number of threats to the environment and humans as a result of their improper use. Adverse reactions include the susceptibility to oxidation in a humid environment (CrCl_2_, FeCl_2_) (change in pT, the risk of explosion during fire), as well as the decomposition of the substance by degassing (MgCl_2_) with the release of HCl at high temperatures, (Na_2_S) decrease in the amount of TCM, a possible corrosion and pressure increase with the separation of highly corrosive H_2_S [13]. Often, in order to improve the thermophysical parameters of salts, mixtures of substances are created, which is why we consider it important to know the reactions with other compounds. Below is a summary of the health and environmental concerns of the salt hydrates discussed (Table 2) based on the analysis of the safety data sheets [18,20,21,22,26,29,30,32].

One of the most commonly used standards for assessing the chemical safety of the substances for human health is the EU CLP Regulation, which entered into force in 2009, adapting the existing EU legislation to the Globally Harmonized System (GHS) [55]. On the basis of the risk level of the substance on the GHS definitions, it can be indicated that magnesium chloride hexahydrate, magnesium nitrate hexahydrate, sodium sulphate decahydrate, sodium acetate trihydrate, and disodium hydrogen phosphate dodecahydrate are substances practically harmless to human health. On the other hand, sodium carbonate decahydrate and calcium chloride hexahydrate, marked in the GHC with the symbol H 319, are medium harmful salts that can cause severe irritation and discomfort. Barium hydroxide octahydrate poses a medium-harm hazard (H302, H314) if ingested and on the skin and may cause permanent damage to organs if inhaled long-term (H332).

## 4. Analysis of the Defects of Salt Hydrates in Terms of Environmental Hazards with an Indication of the Possibility of Minimizing Them

### 4.1. Phase Separation and Supercooling

Supercooling is the first potential disadvantage of salt hydrates. Methods of controlling the supercooling in salt hydrates are known, which allows for stable supercooling, which proves to be an advantage. The authors of [42] write about two different ways of dealing with supercooling. The first is to reduce or avoid the degree of supercooling, and the second is to stabilize the supercooling and use it. The lack of supercooling means that latent heat can be released as soon as the temperature of the salt hydrate reaches its melting point, which is important for the short-term heat storage. Undercooling can be reduced by adding a nucleating agent [23,24,42]. Stable supercooling means that latent heat can be kept at a room temperature for long periods, which is beneficial for the long-term heat storage [42]. Supercooling is closely related to phase segregation, which in turn is caused by different densities of water and salts, as a result of which, during crystallization, forms a precipitate of a solid phase in the water, which does not have the ability to accumulate heat. In order to prevent this phenomenon, intensive mixing is used, nucleation accelerating agents are added, polymers that facilitate crystallization through their cross-linking are used, and thickeners that increase the viscosity are used [56]. Table 3 shows the possibilities of supercooling and the phase separation control in salt hydrates.

#### 4.1.1. MgCl_2_·6H_2_O

Ling et al. [38] believe that the supercooling and phase separation of hydrated inorganic salts severely limits the practical application of magnesium chloride hexahydrate. In addition, the substance easily absorbs water from the air. To prevent this phenomenon, Ling et al. [38] created a composite with graphite carbon nitride (g-C_3_N_4_) with different mass fractions. The results showed that the addition of g-C_3_N_4_ in an amount of 85 wt. reduces the weight change in the magnesium chloride hexahydrate from 5 to 0.4% in two hours. The composite phase change material also has a better thermal stability. Undercooling is eliminated by adding 2% SrCl_2_·6H_2_O. Pilar et al. [57] proved that between the melting and the crystallization of the MgCl_2_-6H_2_O supercooling occurs up to 37 K. The addition of the nucleating substances SrCO_3_, Sr(OH)_2_ or Mg(OH)_2_ suppressed this undesirable phenomenon. In turn, Höhlein et al. [19] obtained supercooling of only 2.8 K with a sample size of 100 g, while large supercooling in the samples of several milligrams was observed.

#### 4.1.2. Mg(NO_3_)_2_·6H_2_O (MNH)

Gupta et al. [37] used five types of carbon materials in the MNH matrix, such as carbon sphere (Sigma Aldrich), graphene nanoflakes (GNPs) (Strem Chemicals), multi-walled carbon nanotubes (MWCNTs) (Ad nanotechnologies), nanographite (NG) (Reinste), and mesoporous carbon (Sigma Aldrich). The optimal mass fraction of the carbon sphere is 0.5% by mass to obtain stable MNH-carbon sphere composites. The carbon sphere acts as a crystallization nucleator. Researchers concluded that the carbon spheres deposited on the MNH matrix showed a better encapsulation and dispersion compared to all other composites due to the smallest differences in shapes and sizes. Carbon spheres can be used as a potential material for solar thermal energy storage systems for various applications as a result of the improvement of the thermal conductivity and heat transfer coefficient. To minimize the risk of phase separation and minimize supercooling, eutectic substances are also created. Ci et al. [58] created a eutectic salt of magnesium nitrate hexahydrate–lithium nitrate in the mass ratio of 85:15, which did not show the phenomenon of phase separation, and the degree of supercooling was less than 1.5 °C. Honcova et al. [59] tested four promising nucleating salts with the addition of 0.5, 1 and 2 wt.% nucleating salt to minimize the MNH overcooling. The results showed that the addition of Mg(OH)_2_, BaO, MgO, and Sr(OH)_2_ suppressed the supercooling below 5 K during 50 melting/crystallization cycles.

#### 4.1.3. Na_2_SO_4_·10H_2_O (SSD)

Zhang et al. [10] proposed a new emulsion polymerization method for encapsulating sodium sulphate decahydrate in silica, which will provide excellent sealing properties. The coating inhibits supercooling and phase separation. In the polymerization process, cyclohexane serves as the oil phase, and the sodium sulphate decahydrate solution is the water phase. In the synthesis of MPCM microcapsules, Triton X-100 was used as an emulsifier. A monomer prepared from tetraethyl orthosilicate (TEOS) and 3-aminopropyltriethoxysilane (APTS) was added to the oil phase to form a coating. Regular spherical microcapsules were obtained, the size of which can be adjusted in the range from 500 nm to 28 μm depending on the amount of Triton X-100 used. Li et al. [39] created a SSD-CBO/EG 7 composite synthesized by SSD, carboxymethyl cellulose (CMC), borax decahydrate, and OP-10. The substance had better thermophysical parameters than pure SSD and a better heat storage efficiency.

#### 4.1.4. CH_3_COONa ·3H_2_O (SAT)

SAT is supercooled well below its normal freezing point. On the one hand, nucleation can be accelerated by the use of special substances (Table 3). On the other hand, there are assumptions about the so-called ‘deactivation’ of the nucleators, especially when the sample temperatures exceed 80 °C. Under such conditions, the nucleation on subsequent cooling becomes sporadic, and when the samples are heated above 90 °C, the nucleation disappears [23]. Oliver et al. [23] believe that SAT, due to the inconsistent melting and nucleus deactivation, is of limited use in heat storage systems, where repeatability over many thousands of heating/cooling cycles and the long-term stability is of key importance. Kumar et al. [25] showed that EG in aqueous SAT increases: the softness of the SAT crystallites, the degree of their supercooling and, most importantly, the effective time of heat release changes by ~10% in relation to the aqueous SAT material. In addition, the maximum water heat dissipation temperature the SAT was adjusted from 56.5 °C to 55 °C, 54.9 °C, 53.5 °C, 51.8 °C, and 43.2 °C using 2%, 3%, 5%, 7%, and 10% by weight, respectively. For example, making the aqueous composite PCM SAT-EG suitable for the desired thermal applications was desirable. Oliver et al. [23] showed that the dilution of the substance with water does not prevent the precipitation of the anhydrous sodium acetate, even at 56% by weight in temperature cycles. Extensive precipitation was observed at 100 cycles. SAT lowers the freezing point and reduces the energy density. Cabeza et al. [62] found that methyl hydroxyethyl cellulose has a load of 30% by weight, which decomposed after heating to a temperature above 65°C, better limiting the phase separation from the starch, bentonite, and methyl cellulose. However, such high loads reduced the latent heat of fusion by up to 35%. Wada et al. [63] improved the performance of the SAT samples by adding polyvinyl alcohol as a thickener in combination with paraffin oil (1%), acetone (0.5%), and additional water (3.5%). However, variability in the measured latent heat of the fusion value was still observed. Wang et al. [42] believe that heat storage using SAT shows good thermal performance and broad prospects as a long-term storage of thermal energy. According to Wang et al. [42], SAT heat storage has a clear advantage over the traditional water-based heat storage. To achieve the same proportion of solar energy, the size of the heat storage using the SAT stable supercooling can be at least 60% smaller than the size of a state-of-the-art hot water tank. This also minimizes the amount of potential waste.

#### 4.1.5. Na_2_CO_3_·10H_2_O (SCD)

Pichandi et al. [44], in order to eliminate the problem of SCD salt melting and inconsistent supercooling, created the eutectic salt sodium carbonate decahydrate (SCD), -Na_2_CO_3_·10H_2_O, and magnesium sulfate heptahydrate (MSH)—MgSO_4_·7H_2_O in the proportions of 70:30. The amount of electricity generated increased by 12.5%. However, the authors found that when the cost of a PV-PCM system is compared to the long-term return on the investment, there is little or no economic benefit, as the PCM material preparation and setup cost extra, even with mass production.

#### 4.1.6. Na_2_HPO_4_·12H_2_O (SD)

Wu et al. [42] encapsulated eutectic (SD) disodium hydrogen phosphate dodecahydrate and sodium carbonate decahydrate in expanded graphite (EG), which showed a positive effect on preventing SD leakage, reducing supercooling (the supercooling rate dropped from 5.7 °C to 0.8 °C) and improving conductivity heat. EG was obtained by heating the expandable graphite at 800 °C for 30 s. SD/EG was obtained by encapsulating the SD in the EG by physical impregnation, and the mass fraction of EG was 15%. Yang et al. [65] developed a CAP/MWCNTs composite that effectively improves the SD phase separation and reduces the supercooling. The CAP is a mixture of sodium carboxymethyl cellulose (CMC), aluminum oxide (Al2O3), and poly(vinylpyrrolidone) (PVP). Using multiwalled carbon nanotubes (MWCNTs), the thermal conductivity was improved, and the temperature of the DHPD-CAP was stabilized at approx. 35.4 °C after 50 melting cycles. Ren et al. [28] prevented leakage of the SD materials by encapsulating them in porous expanded vermiculite (EV). Subsequently, the authors added graphene oxide (GO) to increase the thermal conductivity of the composite.

#### 4.1.7. Ba(OH)_2_·8H_2_O

Barium hydroxide octahydrate, despite its high heat capacity, shows a significant phenomenon of supercooling and phase separation during the cooling process [31]. According to Wang et al. [31], 1% of copper powder, 1% of calcium fluoride, and 1% of calgon can reduce the degree of supercooling to 2.7 °C, 1.8 °C, and 2.3 °C, respectively. The addition of 1% gelatin can prevent phase separation. The problem of leakage in the Ba(OH)_2_·8H_2_O application process can be solved by adding 7% of composite expanded graphite EG [48]. Cui et al. [70] concluded that paraffin (50% by weight) can not only inhibit the evaporation of the crystal water, but also effectively isolate the air by preventing the denaturation of the barium hydroxide octahydrate.

#### 4.1.8. CaCl_2_·6H_2_O

Rezvanpour et al. [50] believe that BaI_2_·6H_2_O, SrCl_2_·6H_2_O and SrBr_2·_6H_2_O are the most effective means of limiting supercooling due to the similarity of the crystal structure to CaCl_2_·6H_2_O. However, the authors point out that there are studies with these substances in which the supercooling was not completely eliminated, possibly due to the low content of the additives. Xu et al. [68] believe that CaCl_2_·2H_2_O plays an important role in reducing the supercooling and can be helpful in adjusting the freezing enthalpy. Xu et al. [69] noticed that adding 0.02 wt.% to the CaCl_2_·6H_2_O. nano-sheet GO, the degree of supercooling is reduced by about 61.6%, In addition, SrCl_2_·6H_2_O 0.8% by weight can reduce the degree of supercooling of the CaCl_2_·6H_2_O sample to approximately 76.5%. The authors developed a novel PCM composite with 0.02 wt. of GO nanosheets and 0.8 wt. CaCl_2_·6H_2_O in which the supercooling decreased by about 99.2%.

### 4.2. Corrosiveness

Corrosiveness is the third most frequently mentioned potential disadvantage of inorganic salt hydrates. An important criterion is that the hydrated salts do not affect the material with which they come into contact. The TCM material should have a specified lifetime of 15–20 years, during which the substance used must retain its properties, and the rate of its decomposition should be as long as possible [71]. Hydrates are also chemically inert towards other construction materials (they cause corrosion) and have a low thermal conductivity [12,72]. Song et al. [12] believe that the corrosiveness of molten salts affects molten salt tanks, pumps, electric heaters, molten salt piping, valves, heat sinks, connecting hoses, flanges, etc. Therefore, a certain amount of chemical corrosion or stress caused by a large difference in temperatures during use may crack the welds of the molten salt tank and lead to the leakage of the substances, thereby degrading the environment. However, Song et al. [12] believe that chloride salts strongly corrode the metal parts of heat accumulation systems faster, but the operating temperature of the chloride salts is as high as 900 °C, which is more economical as a heat carrier and heat storage medium. The ability of salt hydrates to cause corrosion in contact with various materials is presented in the following (Table 4).

Summarizing the information presented in Section 2, Section 3 and Section 4, the inorganic salt hydrates were scored in terms of: their thermophysical properties, economic costs, environmental and human health impacts, phase separation, supercooling, and corrosiveness. Disodium hydrogen phosphate dodecahydrate turned out to be the most prospective salt in terms of the environmental as well as the thermophysical and economic properties for use in solar installations (Table 5).

## 5. Evaluation of the Possibility of Utilizing Used Salts

When designing photovoltaic installations, the goal should be to prevent the generation of waste. According to the Polish Act on Waste [88], this means the measures applied to a product, material, or substance before it becomes waste reduce: (a) the amount of waste, including by the reusing or extending of the period of use of the product, (b) the negative impact of the generated waste on the environment and human health, and (c) the content of harmful substances in the product and material. Therefore, at each stage of installation design, the environmental aspects should be taken into account, with the aim of improving the characteristics of the environmental impact of a given product over its entire life cycle, the so-called environmental performance i.e., ‘eco-design’ [3].

The hydrated salts spent will meet the waste act definition [88]. Waste, in accordance with Art.3, is defined as “a substance which the holder discards, intends to discard or is required to discard”. The waste should be handed over to an entrepreneur who has a permit from the competent authority for waste management or the method of waste disposal should be agreed upon with the relevant Department of Environmental Protection. According to the Regulation of the Minister of Environment of 9 December 2014 on the waste catalogue [89], the unit that generates the waste is required to assign a waste code. The authors proposed the waste code 16 10 Hydrated liquid wastes for the off-site recovery or disposal for the used salt hydrates: 16 10 01—hydrated liquid wastes containing hazardous wastes; 16 10 02—hydrated liquid wastes other than those mentioned in 16 10 01. Currently, the Waste Act [88], Art. 122, prohibits the storage of waste in a landfill 1) in liquid form, including waste containing water in an amount exceeding 95% of the total weight, excluding sludge. Therefore, the generated waste cannot be deposited in liquid waste ponds. The waste with the assumed waste code 16 10 02 can be collected in septic tanks and accepted to the liquid waste collection points operating at water supply and sewage companies [3]. For selected salts, other disposal methods are also listed in their MSDS. However, they apply to pure substances that have not been exposed to contact with the TCM material or to temperature changes. In the case of magnesium nitrate hexahydrate waste, it can be dissolved or mixed with a flammable solvent and burned in a chemical incinerator equipped with an afterburner and scrubber [19]. Similarly, sodium sulphate decahydrate and sodium carbonate decahydrate can be destroyed by burning in specially prepared devices [21,27]. Only the waste codes for two of the salts discussed in the publication are available in the safety data sheets. Note that these are codes for pure substances. For sodium sulphate decahydrate, the waste code is 06 03 14—“Salts and solutions other than those mentioned in 06 03 11 and 06 03 13” [21]. For sodium carbonate decahydrate, the waste code is 16 03 03—“Inorganic waste containing hazardous substances” [27].

## 6. Social Approach to PV Technology with PCM Modules

To the best of our knowledge, no research results have been published so far that would show the public’s approach to solar installations containing PCM modules with inorganic salt hydrates. Thus, photovoltaics are evolving and the society does not always keep up with new technologies, which may cause a negative impact and reduce the demand for this type of installations [2]. As a consequence, this may limit the sustainable development of the environment. According to the Report “Photovoltaic market in Poland 2022” [90], the last 10 years have been called the “golden decade of Polish photovoltaics”. Poland was ranked 2nd in the European Union in terms of the increase in installed PV capacity. Current demand and ever-increasing energy prices will cause PV technologies to constantly develop, and scientists are looking for ways to increase PV efficiency. One of them is an installation with a converter containing salt hydrates. Understanding society’s approach to such solutions becomes crucial to being able to react appropriately and accelerate the development of new technologies.

In order to assess the social aspect, the authors conducted 100 questionnaires using the voluntary selection method. As a rule, this method is considered effective in conducting online surveys, and it gives an overall picture of the society and allows for an initial assessment, which, in the future, may be continued by random selection [91]. Szreder [91] points out that with the increase in information resources on the population subjected to statistical research, the source of information in sample studies is no longer solely a statistical sample. A priori information about the study population becomes an equally important source of information.

The surveys were conducted with the help of the Google Workspace portal. Each participant can only complete one survey. The purpose of the surveys was to learn about society’s approach to the topic of salt hydrates as PCM materials in solar installations. The authors assessed what aspects (technological, economic, and environmental) are important for society when introducing a new panel technology to the market. Public concerns about salt hydrates and their use in photovoltaic installations were also assessed.

Of the 100 completed questionnaires, 54% were women and 46% were men. 91% were people with a higher education and 9% were currently studying. Among the respondents, 50% were people aged 27 to 35 years, 35% were people aged 18 to 26, 12% were people aged 36 to 45, and 3% were aged 46 to 55 years. These were people living in villages and small towns (<50,000 inhabitants) (52%), as well as medium and large cities (>50,000 inhabitants) (48%). An amount of 97% of the respondents were people up to 45 years of age, which was expected by the authors due to the online survey and the more frequent use of this form of communication in this age group. However, the authors consider this to be an advantage. We are convinced that, among young people with a higher education, there is the largest number of recipients of new technologies, sales representatives, and people promoting renewable energy sources. Thus, they are the first link between people on whom attention should be focused when introducing new technologies to the market and striving for sustainable development of the environment. Due to the fact that we believe that the new technology of photovoltaic panels is an opportunity for the sustainable development of municipalities and small towns, the results of the surveys will be considered in two ways.

People living in villages and small towns indicated the economic aspect (installation costs) as the most important aspect when choosing the type of photovoltaic installation (67% of the respondents), followed by the technological aspect (lifetime of the installation and its type) (25%), and the environmental aspect (impact on the environment and human health) was represented by only 8% of the respondents. It is alarming that as many as 33% of the respondents considered the environmental aspect to be of little importance at the installation selection stage. When it comes to the photovoltaic panels supported by converters containing salt hydrates, about 35% of the respondents did not know what salt hydrates were, and as many as 43% were afraid that they may be harmful to human health or were convinced of it. The positive fact is that 41% of people believed that these fears were not so strong that they would not take advantage of the new technology, however, they were currently not convinced about this technology. Considering the environmental aspect, as many as 65% of the respondents were afraid or believed that salt hydrates may have a negative impact on the environment. Much of the worry is borne from a lack of knowledge. An amount of 48% of the people living in small towns and villages at the stage of purchasing the installation did not wonder what will happen to the photovoltaic panels after their lifetime. Thus, extensive promotional and information activities in communities are recommended.

People living in medium and large cities point to economic (59%), technological (25%), and environmental (16%) as the most important aspects when choosing an installation. However, 100% of the respondents believe that the economic aspect is important when choosing the type of installation, and 15% of the respondents believe that the environmental aspect is of little importance. An amount of 16% of people admit that they do not know what salt hydrates are and do not know this type of installation. Respondents are concerned that the use of salt hydrates in the installation may have a negative impact on human health (60%) and the environment (73%). An amount of 48% of people are not convinced whether these fears are strong enough not to make a decision to modernize their fv installation by installing PCM converters. It is also disturbing that 46% of the respondents admit that at the stage of purchasing the installation they do not think about what will happen to the fv panels after their lifetime.

**Figure 1:**
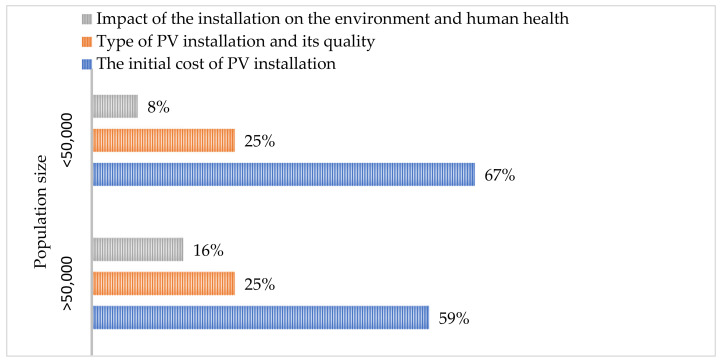
The criteria that residents (depending on the size of the population in which they live) indicate as the most important when purchasing photovoltaic installations.

**Figure 2:**
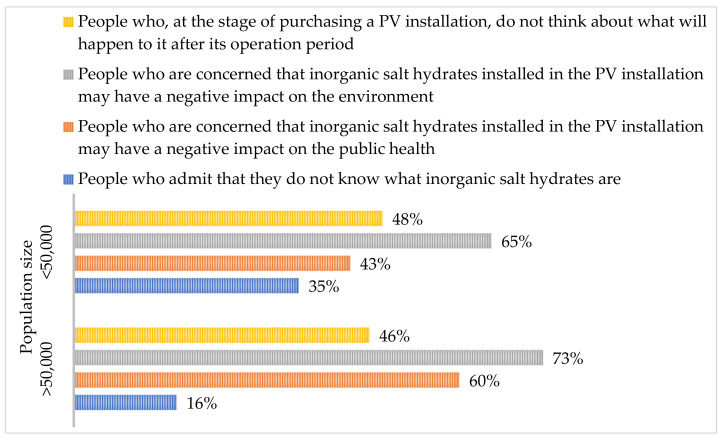
The approach of the society (depending on the size of the population in which they live) to a PV installation with a converter containing inorganic salt hydrates.

Figure 1 and Figure 2 below present a summary of the results of surveys conducted in a group of 100 people for both social groups. From Figure 1, it can be concluded that the main criterion people use when purchasing an installation, regardless of where they live, is initial cost. There was a trend that the residents of smaller towns were slightly more concerned with the initial price of the installation and less concerned with the expected impact of the installation on the environment and human health than the residents of larger cities (>50,000). Furthermore, more than twice as many residents of small towns (33%) than of large towns (16%) considered the environmental aspect to be of little importance when purchasing a PV installation

In Figure 2, the public’s approach to the new technology of PV panels containing a converter with inorganic salt hydrates was assessed.

It is alarming that almost half of the society admits that they do not think about what will happen to the installation after its operation at the stage of purchasing the installation. Furthermore, it has been observed that people living in smaller populations are more than twice as likely (35%/16%) to admit that they do not know what salt hydrates are. It is surprising that inhabitants of larger populations, despite their greater knowledge about salt hydrates, are more concerned about their impact on the environment and human health. Most likely, this knowledge is not enough, and the concerns stem from the general promoted environmental trend in larger cities. Regardless of the social group, such great concerns about the impact of a new technology on the environment and human health may be a barrier to its introduction to the market.

The results of the survey presented in this publication confirm that social acceptance may be a barrier to the implementation of REs and that local acceptance is important, which is consistent with the results obtained by Segreto et al. [92]. According to Segreto et al. [92], an increase in the quality of the environment can be a compensatory measure with a high impact on public opinion. According to our research, society’s approach to environmental issues depends on where you live. In the case of inhabitants of medium and large cities (MC and LC), 15% of them consider the environmental aspect to be of little importance at the stage of choosing a solar installation, while in the case of inhabitants of villages and small towns (V and ST), it is already 33%. According to Segreto et al. [92], the education and income of residents are a key factor influencing social acceptance, and the cost–benefit analysis turned out to be the most influential factor. It is true that our research was carried out on a group of people with higher education, however, we support that the key factor guided by recipients when choosing an installation is its cost (MC and LC 59%/V and ST 67% of respondents). Segreto et al. [92] conclude that contact with installation recipients should be based on trust from planning to development and operation of REs. Many public concerns (economic, environmental, psychological) are based on mistrust and misinformation. The results confirm that much of the worry is caused by lack of knowledge. Thus, Segreto et al. [92] consider it important to provide high-quality technical information, cost–benefit analysis, and environmental impacts. The survey results presented by us support the theses about the lack of sufficient knowledge of installation recipients; however, not all worries can be explained by this factor. Additionally, we noticed some local differences. Only 16% of the residents of medium and large cities admit that they do not know what salt hydrates are, while in the case of the residents of small towns and villages, it is already 35%. Alarmingly, most of the concerns about the impact of solar installations with a converter containing salt hydrates relate to its impact on human health (AC and LC 43%/V and ST 60%) and environmental impact (AC and LC 65%/V and ST 73%).

## 7. Conclusions

This paper presented a comparative overview of the inorganic salt hydrates used to solar installations from environmental and social perspectives. The introduction of new substances in solar installations, the impact of which is unknown in the long term, may pose a serious environmental risk or social dissatisfaction. As a result, there may be efforts to reduce these adverse impacts by selecting appropriate substances. According to the current review of the literature, it has been concluded that:

- Higher values of energy storage density translate into a smaller volume of material necessary to accumulate a given amount of heat, which can minimize the amount of waste. The most advantageous from an economic point of view, due to the relation of price and density, are: calcium chloride hexahydrate, in the first place, and disodium hydrogen phosphate dodecahydrate, in the second place. On the other hand, magnesium chloride hexahydrate and sodium acetate trihydrate seem to be the most unfavorable and can generate the largest volume of waste.
- Based on the risk level of the substance on the GHS definitions, it can be indicated that magnesium chloride hexahydrate, magnesium nitrate hexahydrate, sodium sulfate decahydrate, sodium acetate trihydrate, and disodium hydrogen phosphate dodecahydrate are substances practically harmless to human health. On the other hand, sodium carbonate decahydrate, calcium chloride hexahydrate and barium hydroxide octahydrate are medium-harmful salts.
- Disodium hydrogen phosphate dodecahydrate turned out to be the most promising salt in terms of the thermophysical, economic, and environmental properties for use in solar installations. The waste of this substance is not hazardous waste, and the unfavorable defects (supercooling, phase separation, and corrosiveness) can be easily eliminated, e.g., by encapsulation in expanded graphite. As we believe that it is the most pro-environmental substance, it is advisable to work on the creation of eutectic substances based on disodium hydrogen phosphate dodecahydrate to model its thermophysical properties for various applications.
- Waste code 16 10—hydrated liquid waste is proposed for off-site recovery or disposal for used salt hydrates; 16 10 01—hydrated liquid waste containing hazardous wastes (for magnesium chloride hexahydrate, sodium sulfate decahydrate, sodium acetate trihydrate, sodium carbonate decahydrate, calcium chloride hexahydrate, and barium hydroxide octahydrate); or 16 10 02—hydrated liquid waste other than those mentioned in 16 10 01 (for magnesium nitrate hexahydrate, disodium hydrogen phosphate dodecahydrate.) Waste with the assumed waste code 16 10 02 can be collected in septic tanks and accepted to liquid waste collection points operating in water and wastewater companies.
- The basis for sustainable development is the documentation of salt in terms of all aspects at the same time: technical, economic, environmental, and social. Such activities should be taken into account when designing new technologies in the field of solar installations using inorganic salt hydrates. Further environmental assessments of substances for other uses could be carried out in the future.

Environmental factors seem to be one of the important challenges in introducing new RES technologies that affect public acceptance. However, our research showed that consumers are primarily guided by economic considerations when choosing an installation, and up to 33% of the respondents who live in villages and small towns (<50,000 residents) consider environmental aspects unimportant. Since the main criterion guided by the public is the initial cost of the installation, and the environmental criterion is largely considered unimportant, environmental education in this regard is recommended. We observed that the public’s approach to environmental issues varies according to the size of the locality where they live (<50,000 residents or >50,000 residents). Residents of larger communities are more knowledgeable about inorganic salt hydrates. However, despite their greater knowledge, their concerns about the effects of salt hydrates on human health and the environment are greater than those of residents of smaller towns. Concerns about the possible impact of salt hydrate facilities on society were expressed by 60% of those surveyed, while concerns about their impact on the environment were expressed by 73%. Most likely, there is insufficient knowledge and that the concerns are due to the pro-environmental trend generally promoted in larger cities. In view of this fact, extensive promotion and information activities are recommended for society regarding the technology of panels with salt converters, their impact on the environment, and human health in order to eliminate worries before introducing a new technology to the market.

## Figures and Tables

**Table 1:** Thermophysical parameters of salt hydrates for use in solar installations and their cost.

CAS Number	Salt Hydrates	Melting Temperature °C	Heat of Fusion J/g	Density g/cm^3^	Cost €/kg
7791-18-6	MgCl_2_·6H_2_O	116.7 [18] 115 [19]	166.9 [19]	1.57 [18]	164.9 [20]
Magnesium Chloride Hexahydrate					
13446-18-9	Mg(NO_3_)_2_·6H_2_O	89 [15,21]	162 [15]	1.64 [21]	115.6 [20]
Magnesium Nitrate Hexahydrate					
7727-73-3	Na_2_SO_4_·10H_2_O	32.4 [10,22]	251 [10]	1.46 [22]	104.7 [20]
Sodium Sulfate Decahydrate					
6131-90-4	CH3COONa·3H_2_O	57.9 [23] 58 [24]	260 [25] 250 [26]	1.45 [23]	112.6 [20]
Sodium Acetate Trihydrate					
6132-02-1	Na_2_CO_3_·10H_2_O	30 [27] 32.4 [8]	247 [8]	1.44 [27]	47.4 [20]
Sodium Carbonate Decahydrate					
10039-32-4	Na_2_HPO_4_·12H_2_O	35 [28,29]	256.6 [28] 278.8 [29]	1.52 [28,30]	40.2 [20]
Disodium Hydrogen Phosphate Dodecahydrate					
12230-71-6	Ba(OH)_2_·8H_2_O	78 [31,32]	233–332 [32]	2.18 [31]	127.1 [20]
Barium Hydroxide Octahydrate					
7774-34-7	CaCl_2_·6H_2_O	30 [33] 28.6 [34] 29 [35]	169.5 [34] 160 [35]	1.71 [33] 1.83 [35]	31.4 [20]
Calcium Chloride Hexahydrate					

**Table 2:** The health and environmental concerns of the salt hydrates.

Hydrated Salts	Environmental Hazards	Public Health Hazards
MgCl_2_·6H_2_O	Fire may release hazardous vapours—hydrogen chloride gas, magnesium oxide. Hygroscopic. Corrosive. Hazardous waste (HW).	Use with adequate ventilation.
Mg(NO_3_)_2_·6H_2_O	Avoid strong heating. Decomposition products: magnesium and nitrogen oxides. Hygroscopic. It can cause excessive eutrophication. Increases the flammability of other substances.	Use with adequate ventilation. Dusts may cause irritation of the respiratory and digestive systems. Absorption in the body leads to the formation of methemoglobin.
Na_2_SO_4_·10H_2_O	Fire may release hazardous vapours—sulphur and sodium oxides. Reacts exothermically with strong acids, aluminium and magnesium. Avoid moisture and heating. HW.	Use with adequate ventilation.At a concentration of >500 mg/L of water, it may cause irritation of the digestive tract.
CH_3_COONa·3H_2_O	Fire may release hazardous vapours. Possibility of explosion—reacts with nitrates. Exothermic reaction with fluorine. Hygroscopic. HW.	Use with adequate ventilation. Avoid inhalation of dusts.
Na_2_CO_3_·10H_2_O	Hazardous vapours—carbon and sodium oxides. Hygroscopic. High concentration in water can cause alkalization. Dangerous reactions with aluminium, alkaline earth metals in powder form, organic nitro compounds, fluorine, and non-metal oxides. Violent reactions with sulphuric acid, phosphorus pentoxide, fluorine, lithium, 2,4,6-trinitrotoluene, trichlorethylene, and aluminum. HW.	No special ventilation is required. May cause corneal damage. Irritating to eyes. Eye Irrit. 2, H319.
Na_2_HPO_4_·12H_2_O	Hazardous vapour—phosphorus oxides. Reacts exothermically with strong acids, antipyrine and acetates.	Use with adequate ventilation.
Ba(OH)_2_·8H_2_O	Fire may release hazardous vapours. Barium oxide. Sensitive to air. Reacts with carbon dioxide. Exothermic reaction with hydrogen sulphide and acids. HW.	It is extremely destructive to the respiratory system, eyes, and skin. Corrosive and poisonous to the body. Acute toxicity inhalation H332, dusty H302, corrosive to skin H314.
CaCl_2_·6H_2_O	Dangerous gases—chlorine, hydrogen chloride, chlorine oxides, and calcium oxides. Strongly hygroscopic. Dangerous reactions—boron and calcium oxides, bromine trifluoride; 2-furancarboxylic acid, reacts violently with zinc with evolution of gas; exothermic occurs vinyl ether polymerization reaction catalysis; reacts with water releasing heat. Calcium ions combine with sulphate or carbon ions in the soil to form stable, inorganic salts. Chlorine ions are mobile in the soil. HW.	Use with adequate ventilation. Irritating to eyes. Eye Irrit. 2, H319. Skin irritation with frequent contact.

**Table 3:** Supercooling and the phase segregation of salt hydrates—preventive measures.

Salt Hydrate	Preventive Measure	
	Against Phase Separation	Against Supercooling
MgCl_2_·6H_2_O	85% by weight g-C_3_N_4_ + 2% by weight SrCl_2_·6H_2_O [38]	
		1% by weight SrCO_3_, 0.5% by weight Sr(OH)_2_, Mg(OH)_2_ [57] sufficiently large sample weight 100 g [19]
Mg(NO_3_)_2_·6H_2_O	carbon sphere [37]; Magnesium Nitrate Hexahydrate-Lithium Nitrate eutectic salt 85∶15 mass ratio [58]	
		0.5–2% by weight Mg(OH)_2_, BaO, MgO and Sr(OH)_2_ [59] 3% graphite + 3% graphene [60]
Na_2_SO_4_·10H_2_O	MPCM microcapsules with a silica shell [10]; microcapsules + 1.0 mL Triton X-100 + tetraethyl orthosilicate (TEOS) and 3-aminopropyltriethoxysilane (APTS) [10]; SSD-CBO synthesized by SSD, carboxymethyl cellulose (CMC), borax decahydrate, and OP-10 + 7% by weight EG [39]	
		1–5% by weight nanowires SIC [61]
CH_3_COONa·3H_2_O	methylhydroxyethylcellulose 30% by weight [62]; polyvinyl alcohol combined with paraffin oil (1%), acetone (0.5%) and additional water (3.5%) [63]; SAT adsorbed in expanded vermiculite [62]; poly(methacrylic acid-co-methyl methacrylate (PMMA-co-MMA) 0.8% sodium acetate 57.78%, water 41.42% [23]; sodium salt of poly(methacrylic acid) (Na-PMAA) 0.67%, Sodium Acetate 57.85%, water 41.48% [23]; Sodium Acetate Trihydrate/Disodium Hydrogen Phosphate Dodecahydrate 8.5:1.5 + 1.5% Sodium Metasilicate Nonahydrate [64]	silicon carbide, bentonite, expanded graphite, copper nanoparticles, Disodium Hydrogen Phosphate, Tetrasodium Pyrophosphate [23]; Na_4_P_2_O_7_·10H_2_O and thickening agent polyacrylamide [24]; 2–3% by weight EG in water SAT [25]
Na_2_CO_3_·10H_2_O	Sodium Carbonate Decahydrate (SCD)-Na_2_CO_3_.10H_2_O and Magnesium Sulphate Heptahydrate (MSH)—MgSO_4_·7H_2_O in proportions 70:30 [44].	
		Disodium Hydrogen Phosphate Dodecahydrate and Sodium Carbonate Decahydrate encapsulated into EG [42]
Na_2_HPO_4_·12H_2_O	15% by weight EG [42], mixing Na_2_HPO_4_·12H_2_O, sodium carboxymethyl cellulose (CMC), aluminum oxide (Al_2_O_3_), and poly(vinylpyrrolidone) (PVP) in a mass ratio of 95.8:2:2:0.2 [65],	
	sodium acrylate 3.0–5.0% (by weight), cross-linking agent N,N-methylene bisacrylamide 0.10–0.20% (by weight), K_2_S_2_O_8_ and Na_2_SO_3_ (mass ratio 1:1) 0.06–0.12% (*w*/*w*) [45]; borax, carboxymethyl cellulose (CMC) [45]	
Ba(OH)_2_·8H_2_O	Ba(OH)_2_·8H_2_O/MEG—expanded graphite modified with polyoxyethylene octylphenol ether [66]	
	95.1%Ba(OH)_2_·8H_2_O + 2%Ba(OH)_2_·H_2_O + 2.9%H_2_O [67]; 3% by weight xanthan gum (XG) [47]	1% copper powder, 1% calcium fluoride and 1% calgon (by weight) [31]; 3% calcium fluoride (by weight) [47]
CaCl_2_·6H_2_O		BaI_2_·6H_2_O, SrCl_2_·6H_2_O i SrBr_2_·6H_2_O [50] 66.21% by weight CaCl_2_·2H_2_O [68] 0.5 wt % nano SiO_2_ particles [68] nanosheets GO, SrCl_2_·6H_2_O [69]

**Table 4:** The ability of salt hydrates to cause corrosion.

Salt Hydrates	Corrosiveness of Materials Due to Salt Hydrates	
	Corrosion-Prone Materials	Corrosion-Resistant Materials
MgCl_2_·6H_2_O	copper, aluminium, and stainless steel [73] carbon steel (C 1010); aluminium steel (Al 1100) ^1^ [74]; metal sheets (aluminium, copper, stainless steel) [75]	carbon foam, diatomite, expanded graphite and expanded perlite [46] polyethylene, polyvinyl chloride, unplasticized, butyl rubber ^2^ [75]
Mg(NO_3_)_2_·6H_2_O	mild carbon steel [76]; carbon steel, copper, brass ^3^ [77]	aluminum, SUS316 steel ^3^ [77] mild carbon steel ^4^, aluminium alloy ^5^ [59,60]
Na_2_SO_4_·10H_2_O	Copper ^6^ [78]	aluminum [78]
CH_3_COONa·3H_2_O	brass [42]; copper [42,79,80]; aluminium [42,79]	steel, stainless steel, glass, polyethylene, polypropylene [42]; aluminium [79]
Na_2_CO_3_·10H_2_O	aluminium, copper [80]; plastics (polyacrylates and polysulfide), aluminium, lead, zinc, and zinc brasses [81]	stainless steel, carbon steel, nickel cast iron, nickel and nickel-base alloys, acrylonitrile-butadiene-styrene (ABS), chlorinated polyvinyl chloride (CPVC), nylon, polyethylene, polypropylene, polyvinyl chloride (PVC), Teflon, other fluorocarbons, and some elastomers [81]
Na_2_HPO_4_·12H_2_O	aluminium [79]	carbon foam, diatomaceous earth, expanded graphite, and expanded perlite [82]
Ba(OH)_2_·8H_2_O	copper foam [83]; 20# carbon steel, T2 red copper, H62 brass [84]; aluminium bronze [85]	304 austenitic stainless steel [84]; monel, brass, hastelloy, stainless steel 304, nylon, polypropylene [85]
CaCl_2_·6H_2_O	aluminium (AL 1000 series) [86]; aluminium [87]	stainless steel (SS 347) [86]; carbon foam, diatomite, expanded graphite, and expanded perlite [46]; silver [87]

^1^ 25 wt.% MgCl_2_ solution prepared with 5338 g magnesium chloride hexahydrate mixed with 4662 g tap water. ^2^ MgCl_2_. ^3^ Mg(NO_3_)_2_ 6H_2_O + 10% MgCl_2_ 6H_2_O. ^4^ Mg(NO_3_)_2_ · 6 H_2_O + Ca(NO_3_)_2_ · 4 H_2_O (1:1). ^5^ pure salt hydrate and salt hydrate with 0.5% by weight Mg(OH)_2_ or Sr(OH)_2_. ^6^ E17 Sodium sulphate decahydrate and sodium chloride.

**Table 5:** Summary comparative analysis of 8 inorganic salt hydrates with a view of seven crucial criteria.

Inorganic Salt Hydrates	Criteria									
	T ^a^	C ^b,^**	E ^c^		H ^d^	Ps ^e^	S ^f^		Cr ^g^	Ʃ
Magnesium Chloride Hexahydrate	1	1	1		3	2	2		1	11
Magnesium Nitrate Hexahydrate	1	2	3		3	2	2		2	15
Sodium Sulphate Decahydrate	2	2	2		3	1	2		2	14
Sodium Acetate Trihydrate	3	1	2		3	2	1 (2 *)		2	15
Sodium Carbonate Decahydrate	2	3	1		2	2	2		2	14
Disodium Hydrogen Phosphate Dodecahydrate	3	3	3		3	3	1		2	18
Barium Hydroxide Octahydrate	2	2	2		1	1	1		2	11
Calcium Chloride Hexahydrate	2	3	1		2	2	2		1	13
Criteria	Scale									
	1			2				3		
^a^ T—Thermophysical parameters	medium			good				very good		
^b^ C—Cost **;	high			medium				low		
^c^ E—Adverse impact on the Environment;										
^d^ H—Adverse impact on the Health;										
^e^ Ps—Phase separation;										
^f^ S—Supercooling										
^g^ Cr—Corrosiveness										

* the points have been raised because their high degree of supercooling and high energy storage density make it an ideal flexible material for heat storage [42]. ** the benefit was evaluated from an economic point of view due to the relationship between price and density.

## Data Availability

Not applicable.
